# Baseline T-lymphocyte subset absolute counts can predict both outcome and severity in SARS-CoV-2 infected patients: a single center study

**DOI:** 10.1038/s41598-021-90983-0

**Published:** 2021-06-17

**Authors:** Marco Iannetta, Francesco Buccisano, Daniela Fraboni, Vincenzo Malagnino, Laura Campogiani, Elisabetta Teti, Ilaria Spalliera, Benedetta Rossi, Andrea Di Lorenzo, Raffaele Palmieri, Angela Crea, Marta Zordan, Pietro Vitale, Maria Teresa Voso, Massimo Andreoni, Loredana Sarmati

**Affiliations:** 1 Department of System Medicine, Tor Vergata University, Via Montpellier 1, 00133 Rome, Italy; 2 Infectious Disease Clinic, Policlinico Tor Vergata, Rome, Italy; 3 Department of Biomedicine and Prevention, Tor Vergata University, Rome, Italy; 4 Department of Oncohematology, Policlinico Tor Vergata, Rome, Italy

**Keywords:** Risk factors, Predictive markers, Infection

## Abstract

The aim of this study was to evaluate the role of baseline lymphocyte subset counts in predicting the outcome and severity of COVID-19 patients. Hospitalized patients confirmed to be infected with Severe Acute Respiratory Syndrome Coronavirus 2 (SARS-CoV-2) were included and classified according to in-hospital mortality (survivors/nonsurvivors) and the maximal oxygen support/ventilation supply required (nonsevere/severe). Demographics, clinical and laboratory data, and peripheral blood lymphocyte subsets were retrospectively analyzed. Overall, 160 patients were retrospectively included in the study. T-lymphocyte subset (total CD3+, CD3+ CD4+, CD3+ CD8+, CD3+ CD4+ CD8+ double positive [DP] and CD3+ CD4− CD8− double negative [DN]) absolute counts were decreased in nonsurvivors and in patients with severe disease compared to survivors and nonsevere patients (p < 0.001). Multivariable logistic regression analysis showed that absolute counts of CD3+ T-lymphocytes < 524 cells/µl, CD3+ CD4+ < 369 cells/µl, and the number of T-lymphocyte subsets below the cutoff (T-lymphocyte subset index [TLSI]) were independent predictors of in-hospital mortality. Baseline T-lymphocyte subset counts and TLSI were also predictive of disease severity (CD3+  < 733 cells/µl; CD3+ CD4+ < 426 cells/µl; CD3+ CD8+ < 262 cells/µl; CD3+ DP < 4.5 cells/µl; CD3+ DN < 18.5 cells/µl). The evaluation of peripheral T-lymphocyte absolute counts in the early stages of COVID-19 might represent a useful tool for identifying patients at increased risk of unfavorable outcomes.

## Introduction

In December 2019, a new type of *Betacoronavirus*, identified as Severe Acute Respiratory Syndrome Coronavirus 2 (SARS-CoV-2), emerged in Wuhan, China^1^ and has been associated with the coronavirus disease 2019 (COVID-19)^2^. SARS-CoV-2 has since spread worldwide, causing a pandemic. The clinical manifestations of COVID-19 range from asymptomatic disease to severe respiratory illness^3^.

Lymphocytes play a pivotal role in the immune response against viral infections. Changes in circulating lymphocyte subsets have been described in several respiratory infections caused by RNA-viruses^4–7^.

In SARS-CoV-2 infection, lymphopenia has been consistently reported and associated with COVID-19 severity^8^. T-lymphocyte count reduction has shown promising value as a biomarker for COVID-19 diagnosis and severity prediction^9^. Thus far, several studies on T-lymphocyte subsets in COVID-19 patients have been published, mainly involving the initial Chinese outbreak, but their role in diverse settings has yet to be elucidated.

The aim of this study was to evaluate the role of lymphocyte subset counts in predicting the outcome and severity of SARS-CoV-2 infection using a standardized and reproducible method.

## Methods

### Study design

A single-center, retrospective study involving patients with SARS-CoV-2 infection was performed at the Policlinico Tor Vergata University Hospital of Rome, Italy. All patients were adults (≥ 18 years), hospitalized for COVID-19 in the Infectious Disease Clinic from March 8th to May 7th, 2020, with a positive reverse transcription polymerase chain reaction (RT-PCR) for SARS-CoV-2 on a nasopharyngeal (NPh) swab. Patients with unavailable clinical or laboratory data at baseline were excluded from the analysis.

The study was approved by the Ethics Committee of Fondazione PTV Policlinico Tor Vergata (register number 164/20). All methods were carried out in accordance with relevant guidelines and regulations. The requirement for informed consent was waived by the Ethics Committee considering the retrospective nature of the study, in accordance with local legislation.

### Data collection

An ad hoc electronic database was created to collect demographic data, comorbidities, laboratory findings, oxygen support and ventilation type (either noninvasive or invasive) and outcome (in-hospital mortality). All blood tests were performed in the hospital central laboratory following standard procedures.

### Flow cytometry

T- (CD3+), B- (CD19+), and Natural Killer (NK)-lymphocyte (CD3^neg^CD16 + CD56+) absolute counts were enumerated by multiparametric flow cytometry of peripheral blood (PB). PB samples were processed using a BD FACSDuet™ preparation system integrated with a BD FACSLyric™ flow cytometer. Specimens, as well as reagents, racks and secondary tubes carriers were barcoded to ensure complete traceability of each stage of processing. Lymphocyte subpopulations were assessed using BD Multitest™ 6-color TBNK (FITC-labeled CD3 clone SK7; PE-labeled CD16, clone B73.1, and CD56, clone NCAM 16.2; PerCP-Cy™5.5-labeled CD45, clone 2D1; PE-Cy™7-labeled CD4, clone SK3; APC-labeled CD19, clone SJ25C1; APC-Cy7-labeled CD8, clone SK1) and BD Trucount™ tubes for absolute count with a lyse-no-wash procedure (BD FACS™Lysing Solution). A stabilized blood sample (BD™ Multi-Check Control) was run for each working session. BD FACS Suite™ Clinical software version 1.3 was used to collect and analyze the data (Becton Dickinson Biosciences, San Jose, CA). The BD FACSLyric™ was calibrated on a daily base, using the BD Bioscience™ CS&T Beads to run performance quality controls (Becton Dickinson Biosciences, San Jose, CA). Gating strategy and phenotypic definition of PB lymphocyte subsets are shown in Supplementary Fig. 1.

### Patient classification

Patients were divided into nonsurvivors if death occurred during hospitalization (in-hospital mortality) or survivors who were either: (1) discharged home, (2) moved to a different medical ward or residential structure for COVID-19, being medically stable, because of public health issues, or (3) moved to another hospital and were still alive 30 days after the first hospitalization.

Patients were further stratified into five groups according to the maximal oxygen supply/ventilation support required during the hospitalization: ambient air (AA), Venturi oxygen mask (VMK), nonrebreather oxygen mask with concentrator (NRM), noninvasive ventilation (NIV) and invasive mechanical ventilation through orotracheal intubation (OTI). These categories were additionally grouped into nonsevere (AA and VMK) and severe (NRM, NIV and OTI).

Pneumonia was detected using high-resolution chest CT scan.

### Statistical analysis

Differences between groups were assessed using the Mann–Whitney U test, Kruskal–Wallis test (continuous variable) or Chi^2^ test (categorical variables), as appropriate. Linear correlation was assessed using the Spearman’s correlation test. Univariable and multivariable regression analyses were performed. Cutoff values to differentiate between survivors and nonsurvivors, as well as nonsevere and severe patients, were identified with the receiver operating characteristic (ROC) analysis and confirmed by the Youden’s index. Statistics were performed using JASP (Version 0.11.1. JASP Team, 2019) and Prism 8 for macOS (version 8.2.1. GraphPad Software, San Diego, California USA). A two-sided p value of < 0.05 was considered statistically significant.

The time from symptom onset to the first NPh swab and the first cytofluorimetric assessment was similar between survivors and nonsurvivors, eliminating important confounders, such as diagnosis or hospital admission delay, that did not affect the differences observed in laboratory findings. The interval between NPh and cytofluorimetric assessment was due to the different settings in which the exams were performed: NPh swab in the emergency room and lymphocyte subset characterization upon admission to the infectious disease ward.

## Results

### Study population

**Figure 1:**
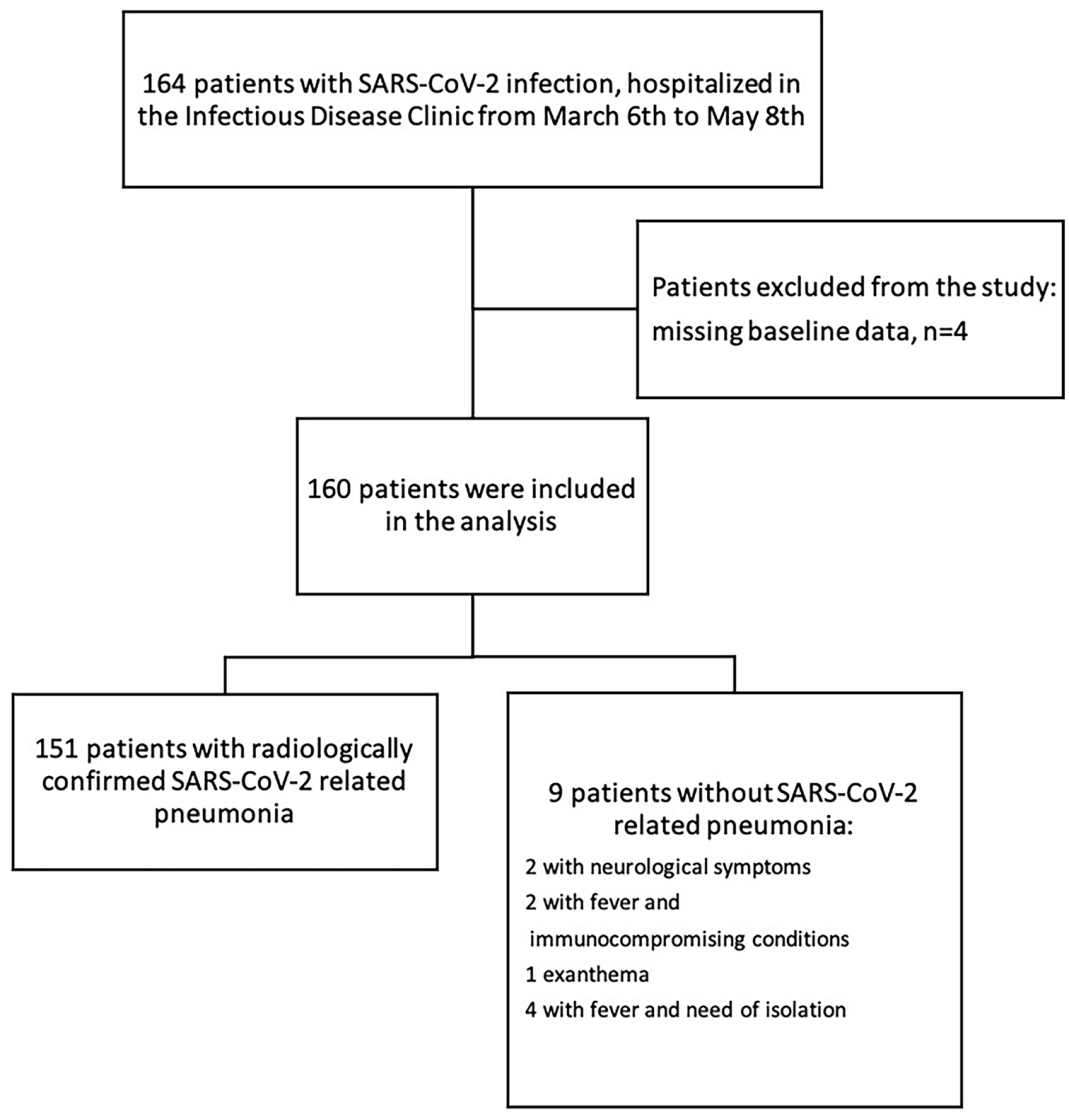
Study population. Flow chart describing the patients included and excluded in the retrospective analysis, with the diagnostic definitions of the 160 patients with confirmed SARS-CoV-2 infection.

One hundred sixty-four consecutive patients hospitalized for COVID-19 from March 8th to May 7th 2020 were enrolled. Four patients were excluded due to missing baseline information (Fig. 1). All patients had at least one confirmed positive molecular test for SARS-CoV-2 RNA detection on a NPh swab. The main clinical condition on admission was SARS-CoV-2-related pneumonia, accounting for 94.4% of the patients (151/160). For the remaining nine patients, hospitalization was due to neurological symptoms (n = 2), fever with immunocompromising condition (n = 2), exanthema (n = 1), or fever with the need for isolation due to public health reasons (n = 4) (Fig. 1). Assessment of peripheral blood lymphocyte subsets was available at baseline for all the included patients. The median age was 62 years, with a prevalence of males (61.2%). The median time from symptom onset to the first positive SARS-CoV-2 NPh swab was 4 days, while the time from symptom onset to lymphocyte subset assessment was 7 days. The majority of the enrolled population (86.3%, 138/160) had at least one comorbidity. Specifically, 50.6% (81/160) had cardiovascular diseases (mainly hypertension), 21.3% (34/160) had neurological/psychiatric disorders (mostly age-related neurocognitive impairment), 16.9% (27/160) had diabetes and 15.6% (25/160) were obese. The rate of Intensive Care Unit (ICU) admission was 16.9% (27/160). The in-hospital mortality rate (nonsurvivors) was 21.3% (34/160) (Table 1).

**Table 1:** Demographic and clinical characteristics of the study population.

	Survivors (N = 126; 78.7%)	Nonsurvivors (N = 34; 21.3%)	p	All patients (N = 160)
Age: median [IQR]	57 [49–71.75]	80 [72.5–85.7]	**< 0.001**	62 [52–78]
Sex: M/F (%)	74/52 (58.7/41.3)	24/10 (70.6/29.4)	0.21	98/62 (61.2/38.8)
Oxygen supply/ventilation support AA/VMK/NRM/NIV/OTI (%)	66/17/16/22/4 (53/14/13/17/3)	1/3/10/5/15 (3/9/29/15/44)	**< 0.001**	68/20/26/27/19 (42/13/16/17/12)
ICU admission (%)	11 (8.8)	16 (47.1)	**< 0.001**	27 (16.9)
Time from symptom onset to 1st NPh-S median days [IQR]	5 [1.25–9]	3 [0–10]	0.281	4 [1–9]
Time from symptom onset to 1st TBNK median days [IQR]	7 [4–12]	7 [4–11]	0.297	7 [4–12]
**Comorbidities**				
Any (%)	104 (82.5)	34 (100)	**0.01**	138 (86.3)
Obesity (%)	21 (16.7)	4 (11.8)	0.475	25 (15.6)
Cardiovascular (%)	54 (42.9)	27 (79.4)	**< 0.001**	81 (50.6)
Diabetes (%)	17 (13.5)	10 (29.4)	**0.029**	27 (16.9)
Endocrinologic (%)	14 (11.1)	2 (5.9)	0.361	16 (10.0)
Cerebrovascular (%)	7 (5.6)	3 (8.8)	0.492	10 (6.3)
Chronic viral hepatitis (%)	2 (1.6)	1 (2.9)	0.610	3 (1.9)
Pulmonary (%)	9 (7.1)	8 (23.5)	**0.006**	17 (10.6)
Renal (%)	7 (5.6)	11 (32.4)	**< 0.001**	18 (11.3)
Solid tumor (%)	12 (9.5)	6 (17.7)	0.189	18 (11.3)
Hematologic (%)	8 (6.4)	5 (14.7)	0.117	13 (8.1)
Neurologic/psychiatric (%)	22 (17.5)	12 (35.3)	**0.026**	34 (21.3)
Rheumatologic (%)	7(5.6)	0 (0)	0.193	7 (4.4)
Other (%)	40 (31.8)	13 (38.2)	0.494	53 (33.1)

Statistically significant p values are highlited in bold.

Differences between groups were assessed using the Mann–Whitney U test (continuous variable) or the Chi^2^ test (categorical variables), as appropriate. A two-sided p value of < 0.05 was considered statistically significant.

*IQR* interquartile range, *AA* ambient air, *VMK* Venturi mask, *NRM* nonrebreather oxygen mask with concentrator, *NIV* noninvasive mechanical ventilation, *OTI* Orotracheal Intubation for mechanical ventilation, *ICU* intensive care unit, *NPh-S* nasopharyngeal swab, *TBNK* T-, B-, Natural Killer-lymphocyte subset assessment.

After stratifying patients according to in-hospital mortality, older age (p < 0.001), ICU admission (p < 0.001), the presence of at least one comorbidity (p = 0.01), cardiovascular comorbidities (p < 0.001), diabetes (p = 0.029), pulmonary comorbidities (p = 0.006), renal impairment (p < 0.001) and neurological/psychiatric disorders (p = 0.026) were associated with in-hospital mortality, at the univariate analysis. Patients with a negative outcome were also more frequently treated with high flux oxygen (NRM) and noninvasive or invasive ventilation (NIV, OTI) compared to survivors (p < 0.001) (Table 1). At the multiple logistic regression analysis, which included age, gender and comorbidities, older age, male sex and pulmonary and renal disease were independent predictors of increased in-hospital mortality (Supplementary Table 2).

### Flow cytometry findings

Baseline relative and absolute counts of circulating B-, T- and NK-lymphocytes were determined. After stratifying patients into survivors and nonsurvivors according to outcome (in-hospital mortality), the latter showed decreased relative and absolute counts of total CD3+ (p < 0.001), CD3 + CD8+ (p < 0.03 and p < 0.001, respectively), CD3 + CD4 + CD8+ double positive (DP) (p = 0.04 and p < 0.001, respectively) and CD3 + CD4 − CD8− double negative (DN) (p < 0.001) T-lymphocytes (Table 2). For CD3 + CD4+ T-lymphocytes, only the absolute counts were significantly reduced in nonsurvivors compared to survivors (p < 0.001). No differences were found in the CD4/CD8 ratio between the two groups. For B-lymphocytes, although the percentage of CD19+ cells was significantly increased in nonsurvivors (p = 0.03), no significant differences were seen in the absolute count between the two groups. NK absolute counts were reduced in nonsurvivors, although at the limit of statistical significance (p = 0.05) (Table 2).

**Table 2:** T-, B- and NK-lymphocyte subpopulation relative and absolute counts at baseline in survivors and nonsurvivors.

	Survivors (N = 126; 78.7%)	Nonsurvivors (N = 34; 21.3%)	p	All patients (N = 160)
Parameter	Median [IQR]	Median [IQR]		Median [IQR]
CD3+ %	73.39 [67.98–79.84]	64.40 [57.96–70.10]	**< 0.001**	72.03 [65.62–79.35]
CD3+ #	825.50 [575.25–1294.25]	397.00 [283.00–626.00]	**< 0.001**	767.00 [480.50–1134.50]
CD3 + CD4+ %	45.34 [37.75–51.17]	40.78 [32.29–47.85]	0.10	44.20 [36.64–50.98]
CD3 + CD4+ #	521.00 [371.50–772.25]	247.00 [195.00–365.00]	**< 0.001**	470.50 [293.00–718.75]
CD3 + CD8+ %	25.00 [18.56–29.79]	22.05 [12.11–26.80]	**0.03**	23.75 [17.25–29.63]
CD3 + CD8+ #	292.50 [168.25–459.50]	127.00 [67.50–210.25]	**< 0.001**	242.00 [137.75–419.00]
CD3 + CD4 + CD8+ %	0.82 [0.55–1.34]	0.71 [0.39–0.92]	**0.04**	0.80 [0.55–1.32]
CD3 + CD4 + CD8+ #	10.00 [6.00–18.00]	4.00 [3.00–6.75]	**< 0.001**	9.00 [5.00–16.00]
CD3 + CD4 − CD8− %	2.73 [1.81–3.83]	1.64 [0.79–2.63]	**< 0.001**	2.49 [1.54–3.61]
CD3 + CD4 − CD8− #	28.50 [18.25–52.75]	12.00 [6.00–20.00]	**< 0.001**	26.00 [15.00–47.00]
CD19+ %	10.45 [7.40–14.46]	13.52 [9.18–22.36]	**0.03**	11.22 [7.51–15.43]
CD19+ #	118.50 [74.50–187.75]	97.00 [64.25–162.25]	0.17	116.00 [70.75–180.75]
CD3^neg^CD16 + CD56+ %	13.11 [8.76–17.95]	16.99 [9.40–27.69]	0.08	13.59 [9.05–19.20]
CD3^neg^CD16 + CD56+ #	145.50 [99.00–226.50]	101.00 [61.00–236.00]	**0.05**	137.00 [91.50–227.75]
CD4/CD8 ratio	1.79 [1.37–2.70]	2.04 [1.17–4.15]	0.32	1.84 [1.34–2.79]

Statistically significant p values are highlited in bold.

Reference values: CD3+ (%): 55–84; CD3+ (cells/µl): 690–2540; CD3 + CD4+ (%): 31–60; CD3 + CD4+ (cells/µl): 410–1590; CD3 + CD8+ (%): 13–41; CD3 + CD8+ (cells/µl): 190–1140; CD19+ (%): 5–25; CD19+ (cells/µl): 90–660; CD3^neg^CD16 + CD56+ (%): 5–27; CD3^neg^CD16 + CD56+ (cells/µl): 90–590; CD4/CD8 ratio: 1.5–2.5

Subpopulation relative counts are expressed as the percentages of total lymphocytes.

Differences between groups were assessed using the Mann–Whitney U test. A two-sided p value of < 0.05 was considered statistically significant.

*IQR* interquartile range.

Given the greater clinical relevance of lymphocyte subset absolute count differences, compared to percentage differentials, further analyses were performed on absolute counts only.

There was an influence of gender on the absolute counts of some T-lymphocyte subsets. Specifically, total CD3+, CD3 + CD4+, CD3 + CD4 + CD8+ and CD3 + CD4 − CD8− absolute counts were reduced in men compared to women (p = 0.037, p = 0.050, p = 0.004 and p = 0.002, respectively). However, when we analyzed separately women and men, all T-lymphocyte subsets were significantly reduced in nonsurvivors, compared to survivors (women: p = 0.013, p = 0.010, p = 0.040, p = 0.003, p = 0.004; men: p < 0.001, p < 0.001, p < 0.001, p < 0.001, p < 0.001, for CD3, CD4, CD8, CD4 + CD8+ DP and CD4 − CD8 − DN, respectively).

Older age was also negatively correlated with decreased absolute counts of CD3, CD4, CD8, CD4 − CD8 − DN and CD19 absolute counts (Spearman’s rho and p-value: − 0.304 and p < 0.001, − 0.280 and p < 0.001, − 0.288 and p < 0.001, − 0.373 and p < 0.001, − 0.174 and p = 0.028).

**Figure 2:**
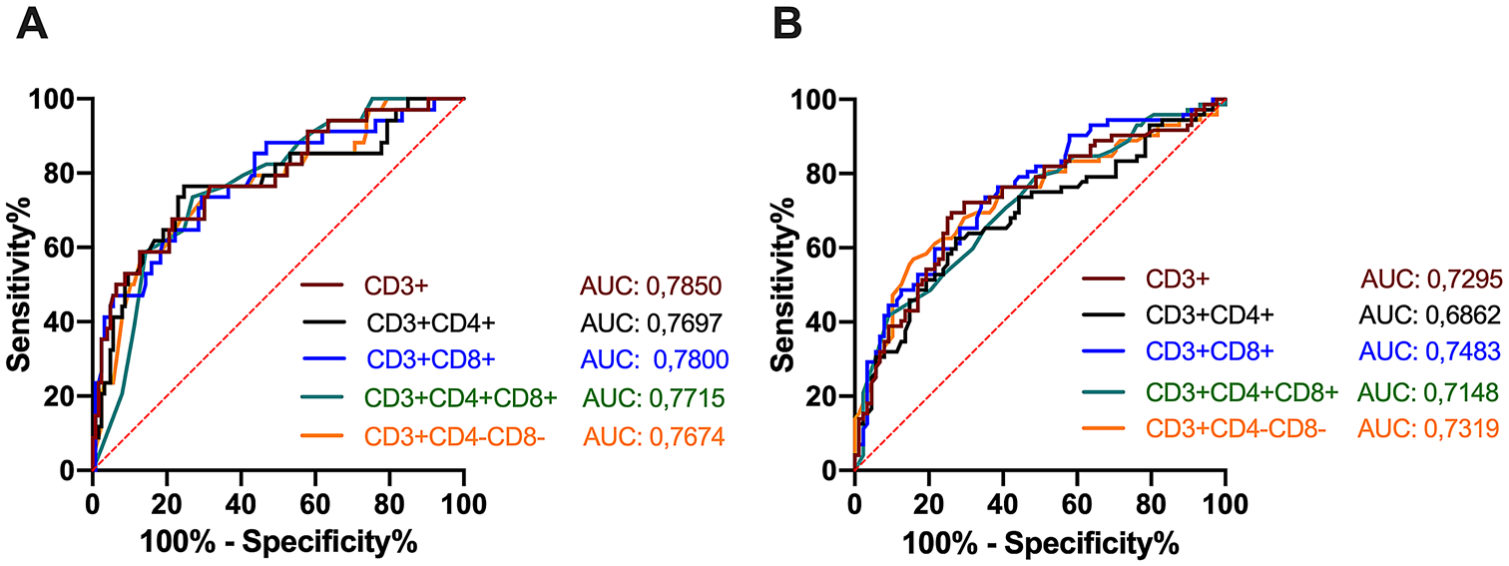
ROC analysis of T-lymphocyte subset absolute counts for in-hospital mortality and disease severity of COVID-19 patients. Receiver operating characteristic (ROC) curves are represented for total CD3+ and four subsets of T lymphocyte cells. Cutoff values were identified with the Youden’s index. (**A**) ROC analysis showing the performance of baseline absolute counts of total T lymphocyte and their subsets in distinguishing fatal cases from survivors. Cutoff values for: CD3+: 524 cells/µl, sensitivity 67,65%, specificity: 78.57%, AUC: 0.7850, p < 0.0001; CD3 + CD4+: < 369 cells/µl, sensitivity 76.47%, specificity: 75.40%, AUC: 0.7697, p < 0.0001; CD3 + CD8+: < 194 cells/µl, sensitivity 73.53%, specificity: 70.63%, AUC: 0.7800, p < 0.0001; CD3 + CD4 + CD8 + DP: < 6.5 cells/µl, sensitivity 73.53%, specificity: 73.02%, AUC: 0.7715, p < 0.0001; CD3 + CD4 − CD8 − DN: < 21.5 cells/µl, sensitivity 76.47%, specificity: 68.25%, AUC: 0.7674, p < 0.0001. (**B**) ROC analysis showing the performance of baseline absolute counts of total T lymphocytes and their subsets in distinguishing severe (NRM, NIV and OTI) from nonsevere (AA and VMK) cases. Cutoff value for: CD3+ : < 733 cells/µl, sensitivity 69.44%, specificity: 73.86%, AUC: 0.7295, p < 0.0001; CD3 + CD4+ : < 426 cells/µl, sensitivity 62.50%, specificity: 72.73%, AUC: 0.6862, p < 0.0001; CD3 + CD8+ : < 262 cells/µl, sensitivity 73.61%, specificity: 64.77%, AUC: 0.7483, p < 0.0001; CD3 + CD4 + CD8 + DP: < 4.5 cells/µl, sensitivity 41.67%, specificity: 90.91%, AUC: 0.7148, p < 0.0001; CD3 + CD4 − CD8 − DN: < 18.5 cells/µl, sensitivity 56.94%, specificity: 84.09%, AUC: 0.7319, p < 0.0001. *AUC* area under the curve, *AA* ambient air, *VMK* Venturi mask, *NRM* nonrebreather oxygen mask with concentrator, *NIV* noninvasive mechanical ventilation, *OTI* Orotracheal Intubation for mechanical ventilation.

The ROC analysis revealed statistically significant cutoff values for T-lymphocyte subset absolute counts that were statistically associated with in-hospital mortality: CD3+ < 524 cells/µl; CD3 + CD4+ < 369 cells/µl; CD3 + CD8+ < 194 cells/µl; CD3 + CD4 + CD8 + DP < 6.5 cells/µl; CD3 + CD4 − CD8 − DN < 21.5 cells/µl (Fig. 2A).

**Figure 3:**
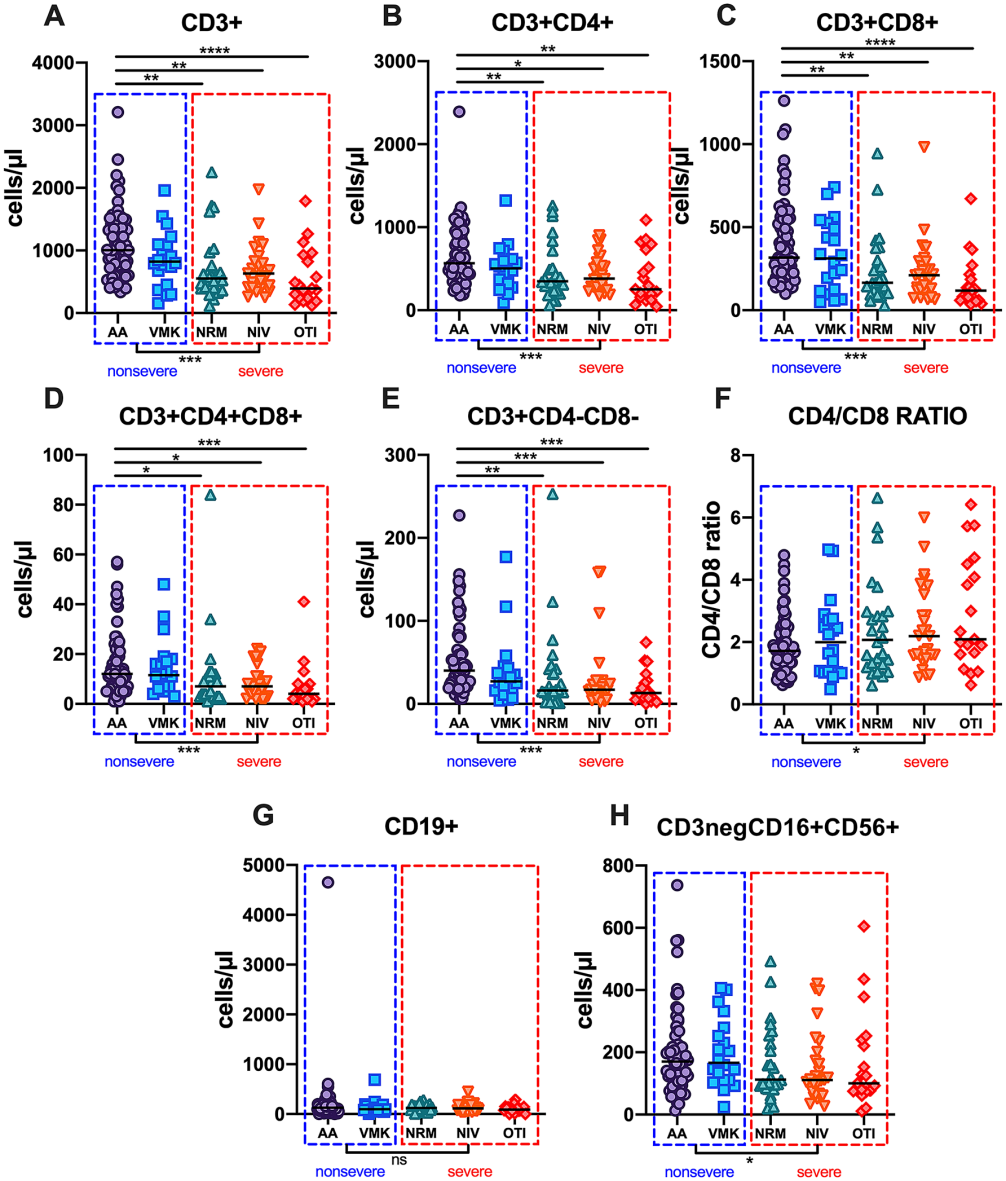
Lymphocyte subset absolute counts in the five groups of patients stratified according to maximal oxygen supply/ventilation support. Absolute counts of total T-lymphocytes (CD3+, (**A**) and related subsets (CD3 + CD4+, (**B**) CD3 + CD8+, (**C**) CD3 + CD4 + CD8 + DP, (**D**) CD3 + CD4 − CD8 − DN, (**E**) CD4/CD8 ratio (**F**) B-lymphocytes (CD19+, (**G**) and NK-cells (CD3^neg^CD16 + CD56+, (**H**) in the study population after stratification according to maximal oxygen supply/ventilation support required during hospitalization. Differences were analyzed using the Kruskal–Wallis and Dunn’s multiple comparison tests; the AA group was used as a reference group for multiple comparisons. Patients were further grouped into nonsevere (AA and VMK, blue dashed line box) and severe (NRM, NIV and OTI, red dashed line box); differences were analyzed using the Mann–Whitney test. A two-sided p value of < 0.05 was considered statistically significant. *AA* ambient air, *VMK* Venturi mask, *NRM* nonrebreather oxygen mask with concentrator, *NIV* noninvasive mechanical ventilation, *OTI* Orotracheal Intubation for mechanical ventilation. *0.01 < p < 0.5; **0.001 < p < 0.01; ***0.0001 < p < 0.00001; ****p < 0.0001.

Considering disease severity, total CD3+, CD3 + CD4+, CD3 + CD8+, CD3 + CD4 + CD8 + DP and CD3 + CD4 − CD8 − DN T-lymphocyte absolute counts progressively decreased from the AA and VMK groups, through the NRM group to the NIV and OTI groups (p < 0.001 for all the T-lymphocyte subset absolute counts) (Table 3, Fig. 3). Posttest analysis with multiple comparison adjustment showed that CD3+, CD3 + CD4+, CD3 + CD8+, CD3 + CD4 + CD8 + DP and CD3 + CD4 − CD8 − DN T-lymphocytes were significantly decreased in the NRM, NIV and OTI groups compared to the AA group, while no differences were found between the VMK and AA groups. No statistically significant differences were observed in the CD4/CD8 ratio or CD19+ and NK-lymphocyte absolute counts (Fig. 3). The NRM group showed median values for T-lymphocyte subsets similar or even inferior to the NIV group. After grouping patients into nonsevere (AA and VMK) and severe (NRM, NIV and OTI), the latter showed a decreased absolute count of total CD3+, CD3 + CD4+, CD3 + CD8+, CD3 + CD4 + CD8 + DP and CD3 + CD4 − CD8 − DN T-lymphocytes (Supplementary Table 1). The ROC analysis revealed statistically significant cutoff values for T-lymphocyte subset absolute counts at baseline, which were able to predict disease severity: CD3+ < 733 cells/µl; CD3 + CD4+ < 426 cells/µl; CD3 + CD8+ < 262 cells/µl; CD3 + CD4 + CD8 + DP < 4.5 cells/µl; CD3 + CD4 − CD8 − DN < 18.5 cells/µl (Fig. 2B).

**Table 3:** T-, B- and NK-lymphocyte subpopulation absolute counts at baseline after patient stratification according to maximal oxygen supply/ventilation support.

	AA (N = 68; 42.5%)	VMK (N = 20; 12.5%)	NRM (N = 26; 16.2%)	NIV (N = 27; 16.9%)	OTI (N = 19; 11.9%)	p
Parameter	Median [IQR]	Median [IQR]	Median [IQR]	Median [IQR]	Median [IQR]	
CD3+ #	1004.00 [742.25–1403.00]	821.00 [620.75–1089.50]	550.50 [405.00–731.75]	633.00 [410.50–810.00]	390.00 [242.00–875.00]	**< 0.001**
CD3 + CD4+ #	565.00 [422.25–869.50]	502.50 [309.25–599.75]	349.00 [236.00–502.25]	382.00 [278.00–554.50]	250.00 [176.00–623.00]	**< 0.001**
CD3 + CD8+ #	316.50 [210.25–551.00]	312.00 [122.75–498.75]	166.00 [96.25–291.00]	211.00 [122.50–279.50]	118.00 [66.00–200.50]	**< 0.001**
CD3 + CD4 + CD8+ #	12.00 [7.00–20.25]	11.50 [6.75–17.25]	7.00 [3.00–11.00]	7.00 [4.00–11.00]	4.00 [2.00–7.00]	** < 0.001**
CD3 + CD4 − CD8− #	40.00 [23.75–59.50]	27.00 [18.25–38.50]	16.00 [11.75–40.75]	17.00 [11.00–27.50]	13.00 [6.50–26.50]	**< 0.001**
CD19 + #	128.50 [72.00–201.50]	97.00 [80.00–177.00]	124.00 [85.25–185.00]	115.00 [63.00–161.00]	90.00 [71.00–133.00]	0.43
CD3^neg^CD16 + CD56+ #	170.50 [104.25–219.00]	166.50 [111.00**–**258.75]	112.00 [87.60**–**221.50]	111.00 [65.00–187.00]	100.00 [75.50–231.00]	0.16
CD4/CD8 ratio	1.72 [1.33–2.41]	2.00 [1.04–2.71]	2.07 [1.30–2.95]	2.19 [1.55–3.19]	2.09 [1.64–4.29]	0.18

Statistically significant p values are highlited in bold.

Reference values: CD3+ (cells/µl): 690–2540; CD3 + CD4+ (cells/µl): 410–1590; CD3 + CD8+ (cells/µl): 190–1140; CD19 + (cells/µl): 90–660; CD3^neg^CD16 + CD56+ (cells/µl): 90–590; CD4/CD8 ratio: 1.5–2.5

Differences between groups were assessed using the Kruskal–Wallis test. A two-sided p value of < 0.05 was considered statistically significant.

*IQR* interquartile range, *AA* ambient air, *VMK* Venturi mask, *NRM* nonrebreather oxygen mask with concentrator, *NIV* noninvasive mechanical ventilation, *OTI* Orotracheal Intubation for mechanical ventilation.

### Laboratory findings

The contribution of other factors to disease severity and in-hospital mortality was evaluated by collecting and analyzing baseline laboratory parameters of the enrolled patients. The results are reported in Table 4. Hemoglobin (Hb, p = 0.014), lymphocyte (Ly, p < 0.001) absolute counts and prothrombin time (PT %, p = 0.03) were significantly decreased in nonsurvivors. Conversely, white blood cell (WBC, p = 0.004) and neutrophil (Neut, p < 0.001) absolute counts, neutrophil-to-lymphocyte ratio (N/L ratio, p < 0.001), C-reactive protein (CRP, p < 0.001), lactate dehydrogenase (LDH, p < 0.001), aspartate transaminase (AST, p = 0.04), creatinine (Crea, p < 0.001), interleukin-6 (IL-6, p < 0.001), D-dimers (p < 0.001), international normalized ratio (INR, p = 0.008), and PT (seconds, p = 0.007) were significantly increased in nonsurvivors (Table 4).

**Table 4:** Laboratory findings at baseline in the study population.

	Survivors (N = 126; 78.7%)	Nonsurvivors (N = 34; 21.3%)	p	All patients (N = 160)
Parameter	Median [IQR]	Median [IQR]		Median [IQR]
Hb (g/dl)	13.10 [11.80–14.30]	11.75 [9.33–13.40]	**0.014**	12.80 [11.10–14.10]
Plt (/µl)	214,000 [171500–283000]	223,000 [138000–260500]	0.55	217.000 [169.000–280.250]
WBC (/µl)	5620 [4160–7130]	7880 [5460–11630]	**0.004**	5975 [4218–7800]
Neut (/µl)	3775 [2425–4960]	6540 [3735–10780]	**< 0.001**	3980 [2533–5910]
Ly (/µl)	1195 [823–1573]	700 [460–930]	**< 0.001**	1110 [728–1472]
N/L ratio	3.29 [1.77–4.77]	10.92 [4.33–15.32]	**< 0.001**	3.73 [2.05–7.19]
CRP (mg/l)	40.75 [12.28–101.63]	101.70 [64.40–138.10]	**< 0.001**	53.10 [18.80–110.40]
LDH (IU/l)	255.00 [212.00–336.00]	356.00 [283.00–513.25]	**< 0.001**	274.00 [217.00**–**349.00]
CPK (IU/l)	88.50 [52.75–173.50]	86.50 [37.25–163.25]	0.71	88.50 [51.25–172.25]
AST (IU/l)	30.00 [23.00–41.00]	34.50 [27.75–73.50]	**0.04**	32.00 [23.00**–**43.00]
ALT (IU/l)	22.00 [15.00–38.00]	22.00 [14.00–39.00]	0.63	22.00 [15.00–38.00]
Conj Bil (mg/dl)	0.33 [0.25–0.43]	0.36 [0.27–0.49]	0.16	0.34 [0.25–0.45]
Total Bil (mg/dl)	0.66 [0.48–0.85]	0.65 [0.51–0.84]	0.91	0.66 [0.49–0.85]
Creatinine (mg/dl)	0.89 [0.77–1.05]	1.35 [0.97–3.18]	**< 0.001**	0.94 [0.78**–**1.17]
IL-6 (pg/ml)	28.30 [12.70–50.55]	48.10 [33.85–100.00]	**< 0.001**	31.45 [14.36**–**56.30]
TNF-alpha (pg/ml)	26.82 [12.49–44.97]	24.65 [16.80–36.33]	0.91	25.81 [12.86–43.68]
d-Dimers (ng/ml)	795.00 [497.00–1533.50]	1554.00 [1043.25–3929.00]	**< 0.001**	997.00 [543.00**–**1765.00]
Fibrinogen (mg/dl)	516.00 [427.00–671.00]	577.50 [437.25–692.00]	0.41	539.00 [429.50–679.00]
INR	1.16 [1.09–1.22]	1.22 1.13–1.32]	**0.008**	1.16 [1.09**–**1.23]
PT (seconds)	14.20 [13.30–14.95]	15.10 [13.90–16.30]	**0.007**	14.30 [13.30**–**15.20]
PT (%)	74.00 [67.00–83.00]	70.00 [62.00–79.75]	**0.03**	73.00 [66.00**–**82.00]

Statistically significant p values are highlited in bold.

Reference values: Hb (g/dl): 12.00–16.00; Plt (/µl): 150,000–450,000; WBC (/µl): 4300–10,800; CRP (mg/l): 0–5.00; LDH (IU/l): 125.00–220.00; CPK (IU/l): 29.00–168.00; AST (IU/l): 5.00–34.00; ALT (IU/l): 0–55.00; Conj Bil (mg/dl): < 0.50; Total Bil (mg/dl): < 1.20; Creatinine (mg/dl): 0.55–1.02; IL-6 (pg/ml): < 50; TNF-alpha (pg/ml): < 12.4; d-Dimers (ng/ml): 0–500.00; Fibrinogen (mg/dl): 200.00–400.00; INR: 0.80–1.20; PT (%): 70.00–130.00.

Differences between groups were assessed using the Mann–Whitney U test (continuous variable). A two-sided p value of < 0.05 was considered statistically significant.

*Hb* hemoglobin, *Plt* platelets, *WBC* white blood cell, *Neut* neutrophils, *Ly* lymphocyte, *N/L ratio* neutrophil-to-lymphocyte ratio, *CRP* C-reactive protein, *LDH* lactate dehydrogenase, *CPK* creatine-phosphokinase, *AST* aspartate transaminase, *ALT* alanine transaminase, *Conj Bil* Conjugated bilirubin, *Total Bil* total bilirubin, *IL-6* interleukin-6, *TNF-*alpha tumor necrosis factor-alpha, *INR* international normalized ratio, *PT* prothrombin time.

For disease severity, the results are shown in Table 5. Patients in the OTI group, compared with the other groups, showed the lowest T-lymphocyte absolute counts (p < 0.001) and the highest values for LDH (p < 0.001), creatinine (p < 0.001) and D-dimers (p < 0.001). WBC (p = 0.035) and neutrophil counts (p < 0.001), N/L ratio (p < 0.001), CRP (p < 0.001) and IL-6 (p < 0.001) were increased in the OTI group, even though the highest values were observed in the NRM group.

**Table 5:** Laboratory parameters at baseline after patient stratification according to maximal oxygen supply/ventilation support.

Parameter	AA (N = 68; 42.5%)	VMK (N = 20; 12.5%)	NRM (N = 26; 16.2%)	NIV (N = 27; 16.9%)	OTI (N = 19; 11.9%)	p
	Median [IQR]	Median [IQR]	Median [IQR]	Median [IQR]	Median [IQR]	
Hb (g/dl)	13.20 [12.28–14.35]	12.300 [11.030–13.300]	12.65 [10.60–13.75]	13.40 [11.00–14.55]	11.900 [9.750–13.500]	0.07
Plt (/µl)	221,500 [175000–288000]	198,000 [165750–265000]	202,500 [122500–259250]	212,000 [160500–281500]	236,000 [138000–277500]	0.60
WBC (/µl)	5330 [4027–7018]	6610 [5110–8320]	7060 [5003–10107]	5610 [4260–6920]	6600 [3760–11210]	**0.035**
Neut (/µl)	3210 [2308–4503]	4680 [3450–7120]	5685 [3913–8865]	4400 [2560–5800]	5280 [2485–8880]	** < 0.001**
Ly (/µl)	1300 [918–1810]	1220 [1000–1460]	840 [692–1353]	960 [670–1230]	630 [430–1150]	** < 0.001**
N/L ratio	2.33 [1.41–3.84]	3.69 [2.32–6.69]	7.32 [3.92–13.07]	3.88 [2.52–9.16]	6.77 [4.15–16.38]	** < 0.001**
CRP (mg/l)	23.05 [10.33–55.48]	90.70 [39.72–141.30]	101.20 [46.45–147.10]	53.40 [17.38–109.65]	77.80 [57.80–125.30]	** < 0.001**
LDH (IU/l)	232.00 [180.00–290.00]	273.00 [226.25–355.25]	333.00 [254.00–415.00]	311.50 [248.00–356.50]	341.00 [283.00–501.50]	** < 0.001**
CPK (IU/l)	76.50 [49.00–152.50]	133.00 [97.00–195.50]	122.00 [49.25–254.00]	83.00 [55.00–191.00]	79.50 [50.50–135.75]	0.16
AST (IU/l)	26.00 [21.00–35.00]	35.00 [24.75–46.25]	38.00 [31.00–42.00]	39.00 [28.00–51.75]	31.00 [24.50–70.50]	**0.015**
ALT (IU/l)	22.00 [16.00–33.00]	17.50 [12.00–37.00]	21.50 [14.00–30.00]	30.00 [14.50–60.75]	24.00 [14.00–43.50]	0.53
Conj Bil (mg/dl)	0.33 [0.24–0.41]	0.32 [0.26–0.45]	0.38 [0.32–0.51]	0.30 [0.23–0.44]	0.36 [0.28–0.47]	0.13
Total Bil (mg/dl)	0.62 [0.48–0.86]	0.65 [0.43–0.83]	0.67 [0.52–0.92]	0.64 [0.49–1.00]	0.66 [0.51–0.81]	0.94
Creatinine (mg/dl)	0.89 [0.77–1.04]	0.99 [0.82–1.77]	1.08 [0.87–1.66]	0.85 [0.73–1.06]	1.22 [0.95–2.31]	** < 0.001**
IL6 (pg/ml)	19.60 [7.82–34.32]	28.75 [18.60–55.25]	46.70 [20.90–90.95]	44.10 [30.10–69.70]	39.05 [21.32–92.65]	** < 0.001**
TNF alpha (pg/ml)	31.96 [12.92–52.92]	17.47 [13.46–31.10]	30.67 [19.05–46.41]	14.81 [10.27–19.78]	24.35 [11.11–34.37]	**0.021**
D-Dimers (ng/ml)	678.50 [410.75–1143.75]	964.00 [649.25–2530.00]	1370.00 [967.00–2980.75]	907.00 [531.50–1867.50]	1636.00 [1060.50–5442.50]	** < 0.001**
Fibrinogen (mg/dl)	467.00 [389.50–604.00]	555.00 [475.00–682.25]	612.50 [454.00–725.00]	597.00 [435.00–692.00]	579.00 [469.00–740.00]	0.064
INR	1.12 [1.08–1.22]	1.19 [1.12–1.28]	1.19 [1.09–1.25]	1.17 [1.08–1.24]	1.22 [1.14–1.27]	0.10
PT (seconds)	13.80 [13.23–15.10]	14.70 [13.78–15.82]	14.55 [13.35–15.35]	14.40 [13.25–15.30]	15.10 [13.95–15.75]	0.08
PT (%)	75.50 [68.00–85.00]	73.50 [60.75–79.00]	71.50 [67.25–78.75]	73.50 [65.25–81.00]	67.00 [62.00–79.50]	0.16

Statistically significant p values are highlited in bold.

Reference values: Hb (g/dl): 12.00–16.00; Plt (/µl): 150,000–450,000; WBC (/µl): 4300–10,800; CRP (mg/l): 0–5.00; LDH (IU/l): 125.00–220.00; CPK (IU/l): 29.00–168.00; AST (IU/l): 5.00–34.00; ALT (IU/l): 0–55.00; Conj Bil (mg/dl): < 0.50; Total Bil (mg/dl): < 1.20; Creatinine (mg/dl): 0.55–1.02; IL-6 (pg/ml): < 50; TNF-alpha (pg/ml): < 12.4; D-Dimers (ng/ml): 0–500.00; Fibrinogen (mg/dl): 200.00–400.00; INR: 0.80–1.20; PT (%): 70.00–130.00.

Differences between groups were assessed using the Kruskal–Wallis test. A two-sided p value of < 0.05 was considered statistically significant.

*Hb* hemoglobin, *Plt* platelets, *WBC* white blood cell, *Neut* neutrophils, *Ly* lymphocyte, *N/L ratio* neutrophil-to-lymphocyte ratio, *CRP* C-reactive protein, *LDH* lactate dehydrogenase, *CPK* creatine-phosphokinase, *AST* aspartate transaminase, *ALT* alanine transaminase, *Conj Bil* Conjugated bilirubin, *Total Bil* total bilirubin, *IL-6* interleukin-6, *TNF-alpha* tumor necrosis factor-alpha, *INR* international normalized ratio, *PT* prothrombin time.

### Logistic regression analysis

Univariable and multivariable logistic regression analyses were performed to assess the mortality- and disease severity-related risk factors. For the univariable analysis, a baseline absolute count below the corresponding cutoff value for each of the T-lymphocyte subsets was statistically associated with an increased risk of in-hospital mortality (total CD3+ < 524 cells/µl: odds ratio (OR) 7.26, p < 0.001; CD3 + CD4+ < 369 cells/µl: OR 9.08, p < 0.001; CD3 + CD8+ < 194 cells/µl: OR 6.34, p < 0.001; CD3 + CD4 + CD8 + DP < 6.5 cells/µl: OR 7.14, p < 0.001; CD3 + CD4 − CD8 − DN < 21.5 cells/µl: OR 6.89, p < 0.001) (Table 6). Additionally, we calculated a “T-lymphocyte subset index” (TLSI), defined as the number of T-lymphocyte subset absolute counts below the cutoff value, ranging from 0 to 4 (all subpopulation absolute counts above or below the cutoffs, respectively). For the univariable logistic regression, TLSI was statistically associated with an increased risk of in-hospital mortality (OR 2.54, p < 0.001) (Table 6). Upon multivariable logistic regression analysis, which included age, gender, number of comorbidities, hemoglobin, WBC absolute counts, N/L ratio, CRP, LDH, AST, creatinine, INR, d-dimer and IL-6 in the model, the independent predictors of increased in-hospital mortality were total CD3+ T-lymphocytes, CD3 + CD4+, and the TLSI, together with age, male gender, LDH and creatinine (Table 6, Supplementary Table 3A–F).

**Table 6:** Univariable and multivariable logistic regression analysis for in-hospital mortality-related risks in patients with SARS-CoV-2 infection.

Parameter	Univariable		Multivariable^a^	
	Odds ratio (CI 95%)	p	Odds ratio (CI 95%)	p
Total CD3+ < 526 cells/µl	7.26 (3.14–16.81)	**< 0.001**	11.57 (1.54–86.77)	**0.017**
CD3 + CD4+ < 369 cells/µl	9.08 (3.71–22.31)	**< 0.001**	8.12 (1.19–55.52)	**0.033**
CD3 + CD8+ < 194 cells/µl	6.34 (2.69–14.94)	**< 0.001**	3.13 (0.57–17.25)	0.19
CD3 + CD4 + CD8+ < 6.5 cells/µl	7.14 (3.02–16.89)	**< 0.001**	4.33 (0.72–26.29)	0.11
CD3 + CD4 − CD8− < 21.5 cells/µl	6.89 (2.85–16.64)	**< 0.001**	1.09 (020–5.84)	0.92
T-lymphocyte subset index (TLSI)^b^	2.54 (1.81–3.56)	**< 0.001**	2.18 (1.00–4.76)	**0.05**

Statistically significant p values are highlited in bold.

Univariable and multivariable logistic regression analyses were performed. Odds ratios and 95% Confidence Interval (CI 95%) are reported. A two-sided p value of < 0.05 was considered statistically significant.

*N/L ratio* neutrophil-to-lymphocyte ratio, *CRP* C-reactive protein, *LDH* lactate dehydrogenase, *AST* aspartate transaminase, *INR* international normalized ratio, *IL-6* interleukin-6.

^a^Adjusting for age, gender, number of comorbidities, hemoglobin, white blood cell absolute counts, N/L ratio, CRP, LDH, AST, Creatinine, INR, d-dimer and IL-6.

^b^The number of T-lymphocyte subset absolute counts under the cut-off value, ranging from 0 to 4.

Interestingly, total T-lymphocyte absolute counts below the cutoff values and all the related subsets, as well as the TLSI, were the only independent predictors of increased risk of disease severity, at the multiple variable logistic regression analysis (Table 7, Supplementary Table 4A–F).

**Table 7:** Univariable and multivariable logistic regression analysis for severity-related risks in patients with SARS-CoV-2 infection.

Parameter	Univariable		Multivariable^a^	
	Odds ratio (CI 95%)	p	Odds ratio (CI 95%)	p
Total CD3+ < 733 cells/µl	6.20 (3.10–12.39)	**< 0.001**	8.12 (2.70–21.17)	**< 0.001**
CD3 + CD4+ < 426 cells/µl	4.54 (2.31–8.91)	**< 0.001**	3.40 (1.27–9.05)	**0.015**
CD3 + CD8+ < 262 cells/µl	4.94 (2.49–9.80)	**< 0.001**	3.65 (1.43–9.34)	**0.007**
CD3 + CD4 + CD8+ < 4.5 cells/µl	7.23 (3.04–17.18)	**< 0.001**	7.08 (2.18–23.02)	**0.001**
CD3 + CD4 − CD8− < 18.5 cells/µl	6.73 (3.21–14.10)	**< 0.001**	5.64 (2.10–15.16)	**< 0.001**
T-lymphocyte subset index (TLSI)^b^	2.23 (1.70–2.92)	**< 0.001**	2.60 (1.66–4.06)	**< 0.001**

Statistically significant p values are highlited in bold.

*N/L ratio* neutrophil-to-lymphocyte ratio, *CRP* C-reactive protein, *LDH* lactate dehydrogenase, *AST* aspartate transaminase, *INR* international normalized ratio, *IL-6* interleukin-6.

Univariable and multivariable logistic regression were performed. Odds ratios and 95% Confidence Interval (CI 95%) are reported. A two-sided p value of < 0.05 was considered statistically significant.

^a^Adjusting for age, gender, number of comorbidities, hemoglobin, white blood cell absolute counts, N/L ratio, CRP, LDH, AST, Creatinine, INR, d-dimer and IL-6.

^b^The number of T-lymphocyte subset absolute counts under the cut-off value, ranging from 0 to 4.

### Inflammatory markers and lymphocyte subpopulations

**Figure 4:**
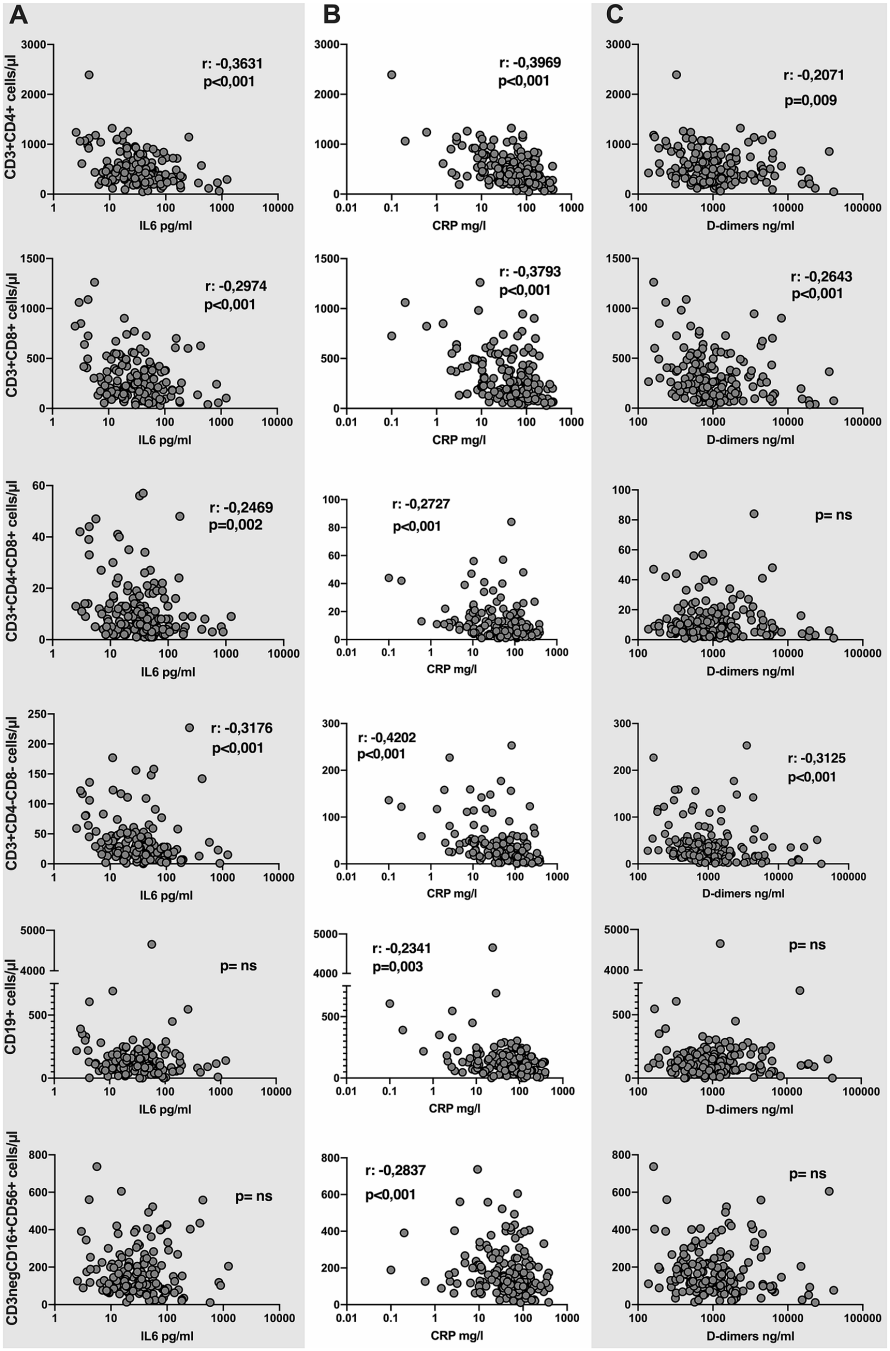
Correlation between lymphocyte subpopulation absolute counts and inflammation markers. Absolute counts of total T-lymphocytes and related subsets (CD3 + CD4+, CD3 + CD8+, CD3 + CD4 + CD8 + DP, CD3 + CD4 − CD8 − DN), B-lymphocytes and NK-cells (CD16 + CD56+) were correlated with IL-6 (column A), CRP serum concentrations (column B) and d-dimer plasma concentration (column C). Correlation was assessed with the Spearman’s test; Spearman r (only if statistically significant) and p are reported in the boxes; a two-sided p value of < 0.05 was considered statistically significant. *ns* not statistically significant, *IL-6* interleukin-6, *CRP* C-reactive protein.

Inflammation parameter (IL-6, TNF-alpha, CRP and d-dimers) correlations with peripheral lymphocyte subsets were assessed using the Spearman’s test (Fig. 4). IL-6 was negatively correlated with CD3 + CD4+, CD3 + CD8+, CD3 + CD4 + CD8 + DP and CD3 + CD4 − CD8 − DN T-lymphocyte absolute counts (p < 0.001, p < 0.001, p = 0.002, p < 0.001, respectively), while no correlation was found with B-lymphocytes or NK-cells. TNF-alpha was not correlated with any of the lymphocyte subsets examined. CRP was negatively correlated with CD3 + CD4+ (p < 0.001), CD3 + CD8+ (p < 0.001), CD3 + CD4 + CD8 + DP (p < 0.001), CD3 + CD4 − CD8 − DN (p < 0.001) T-lymphocyte, B- (p = 0.003) and NK-lymphocyte (p < 0.001) absolute counts. d-dimers were negatively correlated with CD3 + CD4+ (p = 0.009), CD3 + CD8+ (p < 0.001), and CD3 + CD4 − CD8 − DN (p < 0.001) T-lymphocyte absolute counts (Fig. 4).

## Discussion

We retrospectively analyzed the clinical and laboratory parameters of 160 patients with confirmed SARS-CoV-2 infection and showed how T-lymphocyte baseline absolute counts help to predict COVID-19 severity and in-hospital mortality.

As reported in the literature, older age, male sex, the presence of comorbidities, specifically involving pulmonary and renal systems, were independent factors statistically associated with COVID-19 in-hospital mortality^10–12^.

Several studies have shown that lymphopenia is a peculiar finding in SARS-CoV-2-infected patients, even in the early stages of the disease^13,14^. Lymphopenia and N/L ratio have been associated with disease severity, thus allowing for their use as predictive markers of increased risk of in-hospital mortality and ICU admission^8,15^. Our study confirmed these previous results, showing that the baseline lymphocyte absolute count was reduced and the N/L ratio increased in nonsurvivors compared to survivors. We additionally analyzed lymphocyte subsets with more accuracy, demonstrating that the lymphocyte baseline reduction essentially affects the T cell compartment, while B and NK-cells seem to be less influenced by SARS-CoV-2 infection and are not related to disease severity. Interestingly, the baseline reduction of T-lymphocyte absolute count was more pronounced in patients with a more severe disease course, being independent predictors of disease severity. Previous studies have already described the potential role of T-lymphocyte subset absolute counts at baseline to assess the risk of death, progression towards a more severe disease and ICU admission among COVID-19 patients^16–19^. The majority of these studies were conducted in China during the first phase of the COVID-19 pandemic. Studies involving patients from different geographical areas and later during the pandemic are needed to confirm and extend the results from previous works. Baseline T-lymphocyte subset absolute counts were predictive of mortality, while baseline B and NK-cell absolute counts were not associated with COVID-19 mortality. Due to the concomitant reduction of CD4+ and CD8+ T-lymphocyte absolute counts, the CD4/CD8 ratio was not predictive of mortality. He et al. dynamically evaluated peripheral blood lymphocyte subset absolute counts and demonstrated that the nadir of CD4+ and CD8+ T-lymphocytes can persist for several weeks after symptom onset in severe and fatal cases, while a gradual reconstitution is observed in patients who recover^20^. These aspects further confirm the potential application of T-lymphocyte subset absolute counts as a marker of disease severity and fatal outcome, even later during the disease course.

To the best of our knowledge, this is the first study in which CD3 + CD4 + CD8 + DP and CD3 + CD4 − CD8 − DN T-lymphocyte subsets have been considered for clinical purposes. CD3 + CD4 + CD8 + DP T-lymphocytes have been regarded as T-lymphocyte premature precursors, which can be released from the thymus and continue their maturation in the peripheral blood. However, increasing evidence has demonstrated that this subset of DP T-cells represents mature effector memory T-lymphocytes with a T-helper 1/T-cytotoxic 1 profile, and its absolute counts are increased in the peripheral blood of individuals with chronic viral infections^21^. Baseline CD3 + CD4 + CD8 + DP T-lymphocyte absolute counts progressively decreased in patients with more severe COVID-19 disease.

Peripheral CD3 + CD4 − CD8 − DN T-cells are considered regulatory T-lymphocytes, both naïve and antigen-experienced, representing approximately 1–5% of total CD3+ T-cells^22,23^. The role of these cells is still debated, and they have been associated with autoimmune diseases, parasitic and viral infections^24^, such as HIV, where increased absolute counts have been observed in patients with high viral loads^25^. The regulatory effect exerted by this T-lymphocyte subset can also contribute to downregulation of immune-activation through the production of immune modulating cytokines, such as transforming growth factor-beta and IL-10^26^. In our cohort, CD3 + CD4 − CD8 − DN T-lymphocyte absolute counts were significantly reduced in nonsurvivors and progressively decreased from mild to severe COVID-19 patients.

The reduction of peripheral lymphocytes, particularly of T-cell subsets, in COVID-19 patients is still not completely understood. Different phenomena, not mutually exclusive, might be involved: (1) direct infection of T-lymphocyte by SARS-CoV-2 (through the cellular receptor basigin [BSG/CD147]) and consequent cytolysis or apoptosis^27^; (2) lymphocyte migration in the lungs, as assessed by histochemical studies from fatal cases^28,29^; and (3) exhaustion of circulating T-cells of severe COVID-19 patients, characterized by reduced replicative abilities upon stimulation^30,31^. Taken together all these elements can contribute to lymphocyte reduction in the peripheral blood of SARS-CoV-2 infected patients with severe disease.

ROC analysis led to the identification of cutoff values for absolute counts of total CD3+ and T-lymphocyte subsets, which were predictive of disease severity and in-hospital mortality. Our results were similar to those obtained by other authors in different clinical cohorts^20,32,33^. The consistency and comparability of T-lymphocyte subset cutoffs corroborate their utility in clinical practice as prognostic and risk assessment markers in COVID-19 patients. Moreover, the TLSI can represent a useful index with the ability to summarize alterations of different T-lymphocyte subsets, indicating that a higher value of the index is associated with an increased risk of severe disease and in-hospital mortality.

In COVID-19 patients, plasma concentrations of several proinflammatory cytokines and chemokines are extremely increased, characterizing the so-called “cytokine storm”, and they have been associated with disease severity and unfavorable outcomes^3^. In our cohort, we confirmed that baseline IL-6 levels were increased in nonsurvivors compared to survivors and in patients needing high flux oxygen or mechanical ventilation compared to patients with a milder disease course. No differences were found in TNF-alpha plasma levels. Interestingly, serum levels of IL-6 and other inflammatory markers, such as CRP and D-dimers, but not TNF-alpha, were inversely correlated with T-lymphocyte subset absolute counts. Although the p values of the Spearman’s test were highly significant, r coefficients were < 0.5, indicating a weak correlation. Nevertheless, it is relevant that only T-lymphocyte subsets were inversely correlated to those inflammation markers which seem to play a role in the immunopathogenesis of SARS-CoV-2 infection, such as IL-6. These findings confirm the results obtained by other groups in previous works^17,31,33^.

Regarding laboratory baseline parameters, some (WBC and neutrophil counts, N/L ratio, CRP and IL-6 values) showed the most marked alterations in the NRM group, and not in the NIV or OTI groups, as expected. Furthermore, patients in the NRM group showed median values for T-lymphocyte subsets similar or even inferior to the NIV or OTI groups. These aspects could be explained by considering that, although some patients in the NRM group were candidates for mechanical ventilation, either the presence of contraindications or the refusal to allow the procedure led to NRM being continued.

The limitations of this study are its retrospective nature, the single-center design and the absence of a validation prospective cohort. Further studies are needed to confirm our results.

## Conclusion

In conclusion, the most relevant finding of our study is that T-lymphocyte subsets assessed at hospital admission in a standardized and reproducible protocol, are reduced in patients with increased risk of disease progression and unfavorable outcomes.

In the present study, CD3 + CD4 + CD8 + DP and CD3 + CD4 − CD8 − DN “nonconventional” T-lymphocyte subset absolute counts were considered for clinical purpose. Furthermore, the number of T-lymphocyte subset below the cutoff-value (TLSI) was calculated for each patient and considered for predicting disease severity and in-hospital mortality of COVID-19 hospitalized patients.

Specifically, total CD3+ T-lymphocyte, CD3 + CD4+ subset absolute counts and the TLSI were independent predictors of in-hospital mortality, together with older age, male gender, increased LDH and creatinine plasmatic levels, in COVID-19 hospitalized patients.

Total CD3+, all T-lymphocyte subset absolute counts and the TLSI were independent predictors of disease severity in COVID-19 hospitalized patients.

Hence, the assessment of baseline T-lymphocyte subset absolute counts might represent a useful tool for identifying patients with increased risk of disease progression and unfavorable outcomes.

In a global pandemic scenario with limited resources, the possibility of stratifying patients early based on disease severity and outcome risk factors represents a pivotal tool to allocate resources.

## Supplementary Information

Supplementary Figure 1.

Supplementary Tables.

## Data Availability

The data supporting this study will be made available upon reasonable request.
