# Supplementary material for: Baseline T-lymphocyte subset absolute counts can predict both outcome and severity in SARS-CoV-2 infected patients: a single center study
Source: Sci Rep. 2021 Jun 17;11:12762. doi: 10.1038/s41598-021-90983-0 (PMC8211786; doi:10.1038/s41598-021-90983-0)
Supplement: Supplementary file 1 — Supplementary Figure 1. [file 41598_2021_90983_MOESM1_ESM.docx]

**Title**

Baseline T-lymphocyte subset absolute counts can predict both outcome and severity in SARS-CoV-2 infected patients: a single center study

**Authors**

Marco Iannetta^1,2*^, Francesco Buccisano^3^, Daniela Fraboni^4^, Vincenzo Malagnino^1,2^, Laura Campogiani^1,2^, Elisabetta Teti^1,2^, Ilaria Spalliera^1,2^, Benedetta Rossi^1,2^, Andrea Di Lorenzo^1,2^, Raffaele Palmieri^3^, Angela Crea^1,2^, Marta Zordan^1,2^, Pietro Vitale^1,2^, Maria Teresa Voso^3,4^, Massimo Andreoni^1,2^, Loredana Sarmati^1,2^

1 Department of System Medicine, Tor Vergata University, Rome, Italy

2 Infectious Disease Clinic, Policlinico Tor Vergata, Rome Italy

3 Department of Biomedicine and Prevention, Tor Vergata University, Rome, Italy.

4 Department of Oncohematology, Policlinico Tor Vergata, Rome, Italy.

***Corresponding author**

Marco Iannetta, M.D., Ph.D.

Department of System Medicine

Tor Vergata University of Rome

Via Montpellier 1, 00133, Rome, Italy

Email: [marco.iannetta@uniroma2.it](mailto:marco.iannetta@uniroma2.it); mobile: +39 3929986115

**Supplementary Figure 1: Gating strategy for T-, B-, NK-lymphocyte assessment in peripheral blood**


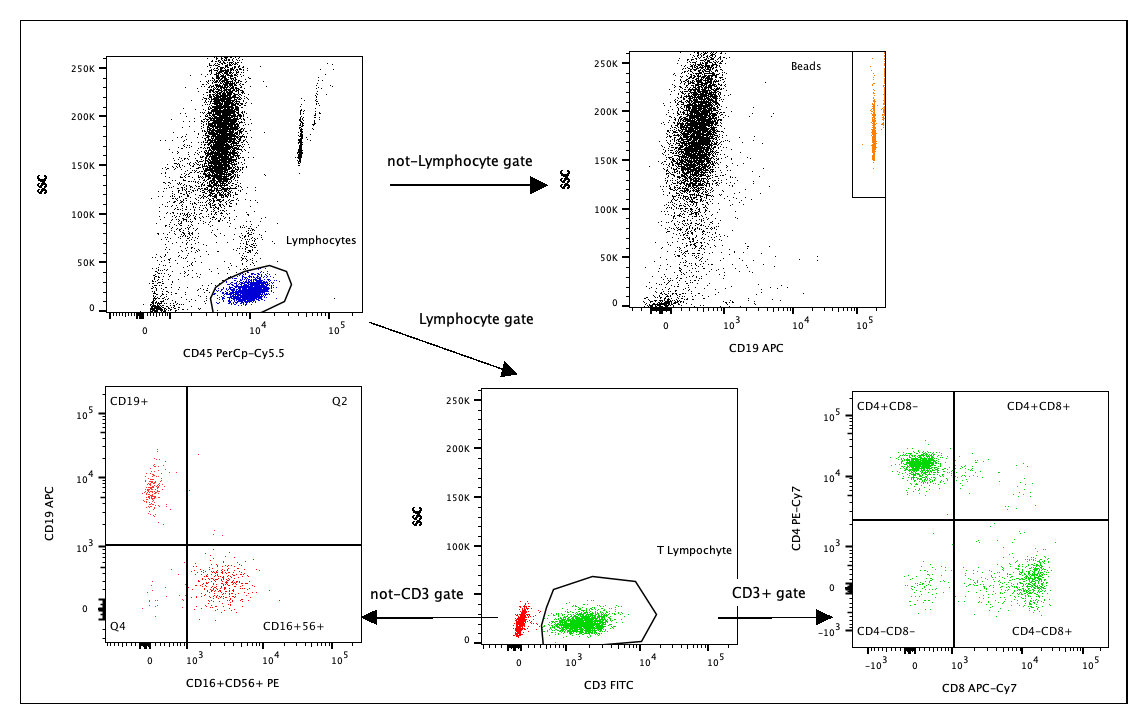


After gating peripheral blood lymphocyte (according to CD45 positivity and low SSC-A parameter, in blue) T-lymphocyte subsets were identified as CD3+ events inside this gate (in green). T-lymphocytes were further characterized according to CD4 and CD8 expression into 4 subsets: CD3+CD4+CD8-; CD3+CD4-CD8+; CD3+CD4-CD8-identified as double negative (DN) and CD3+CD+CD8+ identified as double positive (DP) T-lymphocytes.

Natural Killer (NK)-lymphocyte were identified in the “not-CD3” gate (in red) as CD3-negative cells expressing CD56 and CD16 surface markers (both coupled with the PE fluorochrome).

B lymphocyte were identified in the “not-CD3” gate as CD3-negative cells expressing CD19 surface marker. Beads were identified in the not-lymphocyte gate according to their intense fluorescence in the APC channel and low SSC parameter (in orange).
