# Supplementary material for: Baseline T-lymphocyte subset absolute counts can predict both outcome and severity in SARS-CoV-2 infected patients: a single center study
Source: Sci Rep. 2021 Jun 17;11:12762. doi: 10.1038/s41598-021-90983-0 (PMC8211786; doi:10.1038/s41598-021-90983-0)
Supplement: Supplementary file 2 — Supplementary Tables. [file 41598_2021_90983_MOESM2_ESM.docx]

**Title**

Baseline T-lymphocyte subset absolute counts can predict both outcome and severity in SARS-CoV-2 infected patients: a single center study

**Authors**

Marco Iannetta^1,2*^, Francesco Buccisano^3^, Daniela Fraboni^4^, Vincenzo Malagnino^1,2^, Laura Campogiani^1,2^, Elisabetta Teti^1,2^, Ilaria Spalliera^1,2^, Benedetta Rossi^1,2^, Andrea Di Lorenzo^1,2^, Raffaele Palmieri^3^, Angela Crea^1,2^, Marta Zordan^1,2^, Pietro Vitale^1,2^, Maria Teresa Voso^3,4^, Massimo Andreoni^1,2^, Loredana Sarmati^1,2^

1 Department of System Medicine, Tor Vergata University, Rome, Italy

2 Infectious Disease Clinic, Policlinico Tor Vergata, Rome Italy

3 Department of Biomedicine and Prevention, Tor Vergata University, Rome, Italy.

4 Department of Oncohematology, Policlinico Tor Vergata, Rome, Italy.

***Corresponding author**

Marco Iannetta, M.D., Ph.D.

Department of System Medicine

Tor Vergata University of Rome

Via Montpellier 1, 00133, Rome, Italy

Email: [marco.iannetta@uniroma2.it](mailto:marco.iannetta@uniroma2.it); mobile: +39 3929986115

**Supplementary tables**

**Supplementary Table 1: T-, B- and NK-lymphocyte subpopulation relative and absolute counts at baseline in patients with severe and nonsevere COVID-19**

| Parameter | Nonsevere  (N=88; 55.0%) | Severe  (N=72; 45.0%) | p | All patients  (N=160) |
| --- | --- | --- | --- | --- |
|  | Median [IQR] | Median [IQR] |  | Median [IQR] |
| CD3+ % | 73.65 [68.97-80.04] | 69.26 [62.79-76.38] | **0.001** | 72.03 [65.62-79.35] |
| CD3+ # | 951.00 [710.75-1350.75] | 544.00 [383.75-822.50] | **< 0.001** | 767.00 [480.50-1134.50] |
| CD3+CD4+ % | 44.15 [37.15-51.30] | 44.68 [35.80-50.66] | 0.94 | 44.20 [36.64-50.98] |
| CD3+CD4+ # | 556.00 [385.50-790.25] | 357.00 [232.00-543.75] | **< 0.001** | 470.50 [293.00-718.75] |
| CD3+CD8+ % | 26.34 [20.93-34.00] | 21.44 [13.41-27.31] | **<0.001** | 23.75 [17.25-29.63] |
| CD3+CD8+ # | 316.50 [200.50-543.50] | 158.00 [87.50-267.25] | **< 0.001** | 242.00 [137.75-419.00] |
| CD3+CD4+CD8+ % | 0.87 [0.56-1.35] | 0.71 [0.47-1.16] | 0.053 | 0.80 [0.55-1.32] |
| CD3+CD4+CD8+ # | 12.00 [7.00-19.00] | 6.00 [3.00-11.0] | **< 0.001** | 9.00 [5.00-16.00] |
| CD3+CD4-CD8- % | 2.87 [1.92-3.87] | 1.96 [1.18-3.20] | **0.002** | 2.49 [1.54-3.61] |
| CD3+CD4-CD8- # | 35.50 [22.00-55.00] | 16.50 [7.00-28.00] | **< 0.001** | 26.00 [15.00-47.00] |
| CD19+ % | 10.28 [7.12-13.96] | 12.38 [8.67-18.72] | **0.008** | 11.22 [7.51-15.43] |
| CD19+ # | 119.50 [77.50-194.00] | 112.00 [69.25-166.50] | 0.31 | 116.00 [70.75-180.75] |
| CD3^neg^CD16+CD56+ % | 12.88 [7.99-18.09] | 15.25 [9.37-20.31] | 0.13 | 13.59 [9.05-19.20] |
| CD3^neg^CD16+CD56+ # | 170.50 [108.25-235.50] | 111.00 [75.75-222.50] | **0.013** | 137.00 [91.50-227.75] |
| CD4/CD8 ratio | 1.73 [1.17-2.46] | 2.12 [1.46-3.59] | **0.024** | 1.84 [1.34-2.79] |

IQR: interquartile range. Reference values: CD3+ (%): 55-84; CD3+ (cells/µl): 690-2540; CD3+CD4+ (%): 31-60; CD3+CD4+ (cells/µl): 410-1590; CD3+CD8+ (%): 13-41; CD3+CD8+ (cells/µl): 190-1140; CD19+ (%): 5-25; CD19+ (cells/µl): 90-660; CD3^neg^CD16+CD56+ (%): 5-27; CD3^neg^CD16+CD56+ (cells/µl): 90-590; CD4/CD8 ratio: 1.5-2.5.

Subpopulation relative counts are expressed as the percentages of total lymphocytes.

Differences between groups were assessed using the Mann-Whitney U test (continuous variable). A two-sided p value of <0.05 was considered statistically significant.

**Supplementary Table 2: Multivariable logistic regression analysis for in-hospital mortality-related risks, including age and comorbidities, in patients with SARS-CoV-2 infection**

| **Factors** | **Odds Ratio** | **Lower bound** | **Upper bound** | **p** |
| --- | --- | --- | --- | --- |
| **Age** | **1.072** | **1.013** | **1.124** | **0.004** |
| **Male gender** | **3.601** | **1.050** | **12.355** | **0.042** |
| **Obesity** | 1.157 | 0.233 | 5.745 | 0.858 |
| **Cardiovascular** | 2.045 | 0.598 | 7.000 | 0.254 |
| **Diabetes** | 0.776 | 0.237 | 2.539 | 0.675 |
| **Endocrinologic** | 0.450 | 0.064 | 3.190 | 0.424 |
| **Cerebrovascular** | 1.257 | 0.196 | 8.079 | 0.809 |
| **Chronic viral hepatitis** | 4.919 | 0.139 | 173.816 | 0.381 |
| **Pulmonary** | **4.029** | **1.105** | **14.687** | **0.035** |
| **Renal** | **5.374** | **1.491** | **19.374** | **0.010** |
| **Solid Tumor** | 2.618 | 0.659 | 10.393 | 0.171 |
| **Hematologic** | 2.663 | 0.640 | 11.085 | 0.178 |
| **Neurologic/Psychiatric** | 1.695 | 0.524 | 5.484 | 0.379 |

Multivariable logistic regression analyses were performed. Odds ratios and 95% Confidence Interval (lower bound-upper bound) are reported.

**Supplementary Table 3A: Multivariable logistic regression analysis for in-hospital mortality-related risk, in patients with SARS-CoV-2 infection: CD3**

| **CD3** | **Odds Ratio** | **Lower bound** | **Upper bound** | **p** |
| --- | --- | --- | --- | --- |
| **Age** | **1.149** | **1.045** | **1.263** | **0.004** |
| **Male gender** | **17.195** | **1.498** | **197.386** | **0.022** |
| Comorbidities | 1.580 | 0.803 | 3.111 | 0.186 |
| CRP | 1.000 | 0.990 | 1.011 | 0.984 |
| **LDH** | **1.010** | **1.002** | **1.018** | **0.013** |
| Hb | 0.943 | 0.632 | 1.407 | 0.773 |
| AST | 1.023 | 0.996 | 1.050 | 0.094 |
| **Creatinine** | **6.327** | **1.459** | **27.437** | **0.014** |
| D-Dimer | 1.000 | 1.000 | 1.000 | 0.112 |
| WBC | 0.928 | 0.716 | 1.204 | 0.574 |
| INR | 0.737 | 0.027 | 19.813 | 0.856 |
| IL-6 | 0.997 | 0.991 | 1.003 | 0.359 |
| N/L ratio | 0.907 | 0.772 | 1.065 | 0.233 |
| **CD3<523.5 cells/µl** | **11.572** | **1.543** | **86.772** | **0.017** |

Comorbidities score: number of concomitant chronic disease(s); CRP: C-reactive protein; LDH: lactate dehydrogenase; Hb: hemoglobin; AST: aspartate transaminase; WBC: white blood cell absolute count; INR: international normalized ratio; IL-6: interleukin-6; N/L ratio: neutrophil-to-lymphocyte ratio.

Multivariable logistic regression analyses were performed. Odds ratios and 95% Confidence Interval (lower bound-upper bound) are reported.

**Supplementary Table 3B: Multivariable logistic regression analysis for in-hospital mortality-related risk, in patients with SARS-CoV-2 infection: CD4**

| **CD4** | **Odds Ratio** | **Lower bound** | **Upper bound** | **p** |
| --- | --- | --- | --- | --- |
| **Age** | **1.152** | **1.046** | **1.269** | **0.004** |
| **Male gender** | **21.770** | **1.797** | **263.780** | **0.016** |
| Comorbidities score | 1.521 | 0.761 | 3.040 | 0.236 |
| CRP | 1.000 | 0.990 | 1.011 | 0.955 |
| **LDH** | **1.011** | **1.003** | **1.019** | **0.009** |
| Hb | 0.861 | 0.580 | 1.278 | 0.459 |
| AST | 1.018 | 0.993 | 1.045 | 0.160 |
| **Creatinine** | **5.115** | **1.206** | **21.698** | **0.027** |
| D-Dimer | 1.000 | 1.000 | 1.000 | 0.103 |
| WBC | 0.917 | 0.708 | 1.189 | 0.514 |
| INR | 0.642 | 0.021 | 19.224 | 0.798 |
| IL-6 | 0.997 | 0.991 | 1.003 | 0.391 |
| N/L ratio | 0.922 | 0.790 | 1.075 | 0.300 |
| **CD4<369 cells/µl** | **8.119** | **1.187** | **55.515** | **0.033** |

Comorbidities score: number of concomitant chronic disease(s); CRP: C-reactive protein; LDH: lactate dehydrogenase; Hb: hemoglobin; AST: aspartate transaminase; WBC: white blood cell absolute count; INR: international normalized ratio; IL-6: interleukin-6; N/L ratio: neutrophil-to-lymphocyte ratio.

Multivariable logistic regression analyses were performed. Odds ratios and 95% Confidence Interval (lower bound-upper bound) are reported.

**Supplementary Table 3C: Multivariable logistic regression analysis for in-hospital mortality-related risk, in patients with SARS-CoV-2 infection: CD8**

| **CD8** | **Odds Ratio** | **Lower bound** | **Upper bound** | **p** |
| --- | --- | --- | --- | --- |
| **Age** | **1.123** | **1.027** | **1.228** | **0.011** |
| **Male gender** | **18.495** | **1.846** | **185.356** | **0.013** |
| **Comorbidities** | **2.107** | **1.077** | **4.122** | **0.029** |
| CRP | 1.000 | 0.990 | 1.011 | 0.940 |
| **LDH** | **1.010** | **1.002** | **1.018** | **0.010** |
| Hb | 0.858 | 0.579 | 1.270 | 0.443 |
| AST | 1.017 | 0.992 | 1.043 | 0.182 |
| **Creatinine** | **3.986** | **1.118** | **14.203** | **0.033** |
| D-Dimer | 1.000 | 1.000 | 1.000 | 0.132 |
| WBC | 0.899 | 0.697 | 1.159 | 0.411 |
| INR | 0.773 | 0.037 | 16.050 | 0.868 |
| IL-6 | 0.998 | 0.993 | 1.003 | 0.465 |
| N/L ratio | 0.962 | 0.838 | 1.103 | 0.577 |
| CD8<194 cells/µl | 3.129 | 0.568 | 17.247 | 0.190 |

Comorbidities score: number of concomitant chronic disease(s); CRP: C-reactive protein; LDH: lactate dehydrogenase; Hb: hemoglobin; AST: aspartate transaminase; WBC: white blood cell absolute count; INR: international normalized ratio; IL-6: interleukin-6; N/L ratio: neutrophil-to-lymphocyte ratio. Multivariable logistic regression analyses were performed. Odds ratios and 95% Confidence Interval (lower bound-upper bound) are reported.

**Supplementary Table 3D: Multivariable logistic regression analysis for in-hospital mortality-related risk, in patients with SARS-CoV-2 infection: CD4+CD8+DP**

| **CD4+CD8+DP** | **Odds Ratio** | **Lower bound** | **Upper bound** | **p** |
| --- | --- | --- | --- | --- |
| **Age** | **1.152** | **1.049** | **1.264** | **0.003** |
| **Male gender** | **24.611** | **2.186** | **277.042** | **0.010** |
| Comorbidities | 1.876 | 0.986 | 3.572 | 0.055 |
| CRP | 1.003 | 0.992 | 1.013 | 0.620 |
| **LDH** | **1.009** | **1.002** | **1.017** | **0.018** |
| Hb | 0.907 | 0.609 | 1.350 | 0.630 |
| AST | 1.020 | 0.995 | 1.046 | 0.114 |
| **Creatinine** | **4.094** | **1.085** | **15.455** | **0.038** |
| D-Dimer | 1.000 | 1.000 | 1.000 | 0.084 |
| WBC | 0.929 | 0.712 | 1.213 | 0.590 |
| INR | 0.782 | 0.035 | 17.548 | 0.877 |
| IL-6 | 0.998 | 0.992 | 1.004 | 0.610 |
| N/L ratio | 0.921 | 0.786 | 1.080 | 0.312 |
| CD4+CD8+<6,5 cells/µl | 4.334 | 0.715 | 26.290 | 0.111 |

Comorbidities score: number of concomitant chronic disease(s); CRP: C-reactive protein; LDH: lactate dehydrogenase; Hb: hemoglobin; AST: aspartate transaminase; WBC: white blood cell absolute count; INR: international normalized ratio; IL-6: interleukin-6; N/L ratio: neutrophil-to-lymphocyte ratio.

Multivariable logistic regression analyses were performed. Odds ratios and 95% Confidence Interval (lower bound-upper bound) are reported.

**Supplementary Table 3E: Multivariable logistic regression analysis for in-hospital mortality-related risk, in patients with SARS-CoV-2 infection: CD4-CD8-DN**

| **CD4-CD8-DN** | **Odds Ratio** | **Lower bound** | **Upper bound** | **p** |
| --- | --- | --- | --- | --- |
| **Age** | **1.142** | **1.043** | **1.251** | **0.004** |
| **Male gender** | **26.302** | **2.200** | **314.410** | **0.010** |
| **Comorbidities** | **1.959** | **1.024** | **3.745** | **0.042** |
| CRP | 1.001 | 0.991 | 1.011 | 0.856 |
| **LDH** | **1.011** | **1.003** | **1.018** | **0.006** |
| Hb | 0.865 | 0.595 | 1.258 | 0.449 |
| AST | 1.018 | 0.993 | 1.043 | 0.156 |
| **Creatinine** | **4.654** | **1.205** | **17.975** | **0.026** |
| D-Dimer | 1.000 | 1.000 | 1.000 | 0.113 |
| WBC | 0.847 | 0.662 | 1.083 | 0.186 |
| INR | 0.758 | 0.042 | 13.635 | 0.851 |
| IL-6 | 0.998 | 0.993 | 1.004 | 0.539 |
| N/L ratio | 0.988 | 0.868 | 1.124 | 0.849 |
| CD4-CD8-<21,5 cells/µl | 1.090 | 0.203 | 5.846 | 0.920 |

Comorbidities score: number of concomitant chronic disease(s); CRP: C-reactive protein; LDH: lactate dehydrogenase; Hb: hemoglobin; AST: aspartate transaminase; WBC: white blood cell absolute count; INR: international normalized ratio; IL-6: interleukin-6; N/L ratio: neutrophil-to-lymphocyte ratio.

Multivariable logistic regression analyses were performed. Odds ratios and 95% Confidence Interval (lower bound-upper bound) are reported.

**Supplementary Table 3F: Multivariable logistic regression analysis for in-hospital mortality-related risk, in patients with SARS-CoV-2 infection: TLSI**

| **TLSI** | **Odds Ratio** | **Lower bound** | **Upper bound** | **p** |
| --- | --- | --- | --- | --- |
| **Age** | **1.133** | **1.033** | **1.242** | **0.008** |
| **Male gender** | **13.838** | **1.191** | **160.809** | **0.036** |
| Comorbidities | 1.820 | 0.936 | 3.539 | 0.078 |
| CRP | 1.001 | 0.991 | 1.011 | 0.872 |
| LDH | 1.008 | 1.000 | 1.017 | 0.052 |
| Hb | 0.874 | 0.590 | 1.295 | 0.502 |
| AST | 1.023 | 0.994 | 1.053 | 0.117 |
| Creatinine | 3.731 | 0.963 | 14.456 | 0.057 |
| D-Dimer | 1.000 | 1.000 | 1.000 | 0.111 |
| WBC | 0.977 | 0.743 | 1.285 | 0.867 |
| INR | 0.975 | 0.053 | 17.806 | 0.986 |
| IL-6 | 0.998 | 0.992 | 1.004 | 0.463 |
| N/L ratio | 0.904 | 0.765 | 1.069 | 0.240 |
| **TLSI** | **2.184** | **1.002** | **4.764** | **0.050** |

Comorbidities score: number of concomitant chronic disease(s); CRP: C-reactive protein; LDH: lactate dehydrogenase; Hb: hemoglobin; AST: aspartate transaminase; WBC: white blood cell absolute count; INR: international normalized ratio; IL-6: interleukin-6; N/L ratio: neutrophil-to-lymphocyte ratio; TLSI: T-lymphocyte subset index, representing the number of T-lymphocyte subset absolute counts under the cut-off value, ranging from 0 to 4.

Multivariable logistic regression analyses were performed. Odds ratios and 95% Confidence Interval (lower bound-upper bound) are reported.

**Supplementary Table 4A: Multivariable logistic regression analysis for disease severity-related risk, in patients with SARS-CoV-2 infection: CD3**

| **CD3** | **Odds Ratio** | **Lower bound** | **Upper bound** | **p** |
| --- | --- | --- | --- | --- |
| Age | 1.152 | 0.978 | 1.046 | 0.509 |
| Male gender | 0.922 | 0.828 | 5.924 | 0.113 |
| Comorbidities | 0.997 | 0.846 | 1.787 | 0.278 |
| CRP | 21.770 | 0.993 | 1.007 | 0.968 |
| LDH | 1.521 | 0.998 | 1.008 | 0.212 |
| Hb | 1.000 | 0.994 | 1.027 | 0.212 |
| AST | 1.011 | 0.706 | 1.316 | 0.817 |
| Creatinine | 0.861 | 1.000 | 1.000 | 0.248 |
| D-Dimer | 1.018 | 0.709 | 1.126 | 0.339 |
| WBC | 5.115 | 0.825 | 1.200 | 0.960 |
| INR | 1.000 | 0.031 | 1.500 | 0.121 |
| IL-6 | 0.917 | 0.998 | 1.009 | 0.276 |
| N/L ratio | 0.642 | 0.866 | 1.078 | 0.537 |
| **CD3<733 cells/µl** | **8.119** | **2.700** | **21.166** | **< 0.001** |

Comorbidities score: number of concomitant chronic disease(s); CRP: C-reactive protein; LDH: lactate dehydrogenase; Hb: hemoglobin; AST: aspartate transaminase; WBC: white blood cell absolute count; INR: international normalized ratio; IL-6: interleukin-6; N/L ratio: neutrophil-to-lymphocyte ratio.

Multivariable logistic regression analyses were performed. Odds ratios and 95% Confidence Interval (lower bound-upper bound) are reported.

**Supplementary Table 4B: Multivariable logistic regression analysis for disease severity-related risk, in patients with SARS-CoV-2 infection: CD4**

| **CD4** | **Odds Ratio** | **Lower bound** | **Upper bound** | **p** |
| --- | --- | --- | --- | --- |
| Age | 1.016 | 0.984 | 1.050 | 0.321 |
| Male gender | 2.182 | 0.850 | 5.603 | 0.105 |
| Comorbidities | 1.252 | 0.878 | 1.785 | 0.215 |
| CRP | 0.999 | 0.993 | 1.006 | 0.814 |
| LDH | 1.004 | 0.999 | 1.008 | 0.125 |
| Hb | 0.904 | 0.723 | 1.129 | 0.372 |
| AST | 1.007 | 0.990 | 1.024 | 0.416 |
| Creatinine | 0.988 | 0.728 | 1.341 | 0.937 |
| D-Dimer | 1.000 | 1.000 | 1.000 | 0.195 |
| WBC | 0.923 | 0.772 | 1.103 | 0.377 |
| INR | 0.232 | 0.034 | 1.602 | 0.138 |
| IL-6 | 1.003 | 0.998 | 1.008 | 0.282 |
| N/L ratio | 1.007 | 0.905 | 1.120 | 0.905 |
| **CD4<426 cells/µl** | **3.395** | **1.274** | **9.046** | **0.015** |

Comorbidities score: number of concomitant chronic disease(s); CRP: C-reactive protein; LDH: lactate dehydrogenase; Hb: hemoglobin; AST: aspartate transaminase; WBC: white blood cell absolute count; INR: international normalized ratio; IL-6: interleukin-6; N/L ratio: neutrophil-to-lymphocyte ratio.

Multivariable logistic regression analyses were performed. Odds ratios and 95% Confidence Interval (lower bound-upper bound) are reported.

**Supplementary Table 4C: Multivariable logistic regression analysis for disease severity-related risk, in patients with SARS-CoV-2 infection: CD8**

| **CD8** | **Odds Ratio** | **Lower bound** | **Upper bound** | **p** |
| --- | --- | --- | --- | --- |
| Age | 1.014 | 0.981 | 1.047 | 0.411 |
| Male gender | 2.116 | 0.822 | 5.448 | 0.120 |
| Comorbidities | 1.322 | 0.925 | 1.889 | 0.125 |
| CRP | 0.998 | 0.992 | 1.004 | 0.554 |
| LDH | 1.003 | 0.998 | 1.008 | 0.196 |
| Hb | 0.878 | 0.704 | 1.096 | 0.250 |
| AST | 1.010 | 0.993 | 1.027 | 0.264 |
| Creatinine | 0.959 | 0.707 | 1.301 | 0.788 |
| D-Dimer | 1.000 | 1.000 | 1.000 | 0.258 |
| WBC | 0.927 | 0.775 | 1.108 | 0.405 |
| INR | 0.224 | 0.035 | 1.435 | 0.114 |
| IL-6 | 1.003 | 0.998 | 1.009 | 0.216 |
| N/L ratio | 1.024 | 0.927 | 1.131 | 0.640 |
| **CD8<262 cells/µl** | **3.651** | **1.427** | **9.338** | **0.007** |

Comorbidities score: number of concomitant chronic disease(s); CRP: C-reactive protein; LDH: lactate dehydrogenase; Hb: hemoglobin; AST: aspartate transaminase; WBC: white blood cell absolute count; INR: international normalized ratio; IL-6: interleukin-6; N/L ratio: neutrophil-to-lymphocyte ratio.

Multivariable logistic regression analyses were performed. Odds ratios and 95% Confidence Interval (lower bound-upper bound) are reported.

**Supplementary Table 4D: Multivariable logistic regression analysis for disease severity-related risk, in patients with SARS-CoV-2 infection: CD4+CD8+DP**

| **CD4+CD8+DP** | **Odds Ratio** | **Lower bound** | **Upper bound** | **p** |
| --- | --- | --- | --- | --- |
| Age | 1.010 | 0.978 | 1.044 | 0.530 |
| Male gender | 2.053 | 0.775 | 5.435 | 0.148 |
| **Comorbidities** | **1.453** | **1.002** | **2.107** | **0.049** |
| CRP | 1.001 | 0.995 | 1.008 | 0.723 |
| LDH | 1.003 | 0.998 | 1.007 | 0.263 |
| Hb | 0.871 | 0.698 | 1.087 | 0.221 |
| AST | 1.011 | 0.994 | 1.029 | 0.204 |
| Creatinine | 0.915 | 0.663 | 1.265 | 0.593 |
| D-Dimer | 1.000 | 1.000 | 1.000 | 0.108 |
| WBC | 0.951 | 0.792 | 1.142 | 0.592 |
| INR | 0.335 | 0.043 | 2.588 | 0.294 |
| IL-6 | 1.003 | 0.998 | 1.008 | 0.184 |
| N/L ratio | 0.999 | 0.898 | 1.111 | 0.983 |
| **CD4+CD8+<4.5 cells/µl** | **7.078** | **2.176** | **23.022** | **0.001** |

Comorbidities score: number of concomitant chronic disease(s); CRP: C-reactive protein; LDH: lactate dehydrogenase; Hb: hemoglobin; AST: aspartate transaminase; WBC: white blood cell absolute count; INR: international normalized ratio; IL-6: interleukin-6; N/L ratio: neutrophil-to-lymphocyte ratio.

Multivariable logistic regression analyses were performed. Odds ratios and 95% Confidence Interval (lower bound-upper bound) are reported.

**Supplementary Table 4E: Multivariable logistic regression analysis for disease severity-related risk, in patients with SARS-CoV-2 infection: CD4-CD8-DN**

| **CD4-CD8-DN** | **Odds Ratio** | **Lower bound** | **Upper bound** | **p** |
| --- | --- | --- | --- | --- |
| Age | 1.005 | 0.973 | 1.039 | 0.754 |
| Male gender | 1.922 | 0.719 | 5.137 | 0.193 |
| Comorbidities | 1.305 | 0.912 | 1.866 | 0.145 |
| CRP | 0.997 | 0.991 | 1.004 | 0.417 |
| LDH | 1.003 | 0.998 | 1.008 | 0.204 |
| Hb | 0.856 | 0.683 | 1.074 | 0.179 |
| AST | 1.011 | 0.994 | 1.029 | 0.210 |
| Creatinine | 0.898 | 0.652 | 1.238 | 0.512 |
| D-Dimer | 1.000 | 1.000 | 1.000 | 0.378 |
| WBC | 0.895 | 0.747 | 1.074 | 0.232 |
| INR | 0.331 | 0.057 | 1.919 | 0.217 |
| IL-6 | 1.004 | 0.998 | 1.009 | 0.157 |
| N/L ratio | 1.047 | 0.946 | 1.158 | 0.376 |
| **CD4-CD8-<18.5 cells/µl** | **5.637** | **2.096** | **15.160** | **< 0.001** |

Comorbidities score: number of concomitant chronic disease(s); CRP: C-reactive protein; LDH: lactate dehydrogenase; Hb: hemoglobin; AST: aspartate transaminase; WBC: white blood cell absolute count; INR: international normalized ratio; IL-6: interleukin-6; N/L ratio: neutrophil-to-lymphocyte ratio.

Multivariable logistic regression analyses were performed. Odds ratios and 95% Confidence Interval (lower bound-upper bound) are reported.

**Supplementary Table 4F: Multivariable logistic regression analysis for disease severity-related risk, in patients with SARS-CoV-2 infection: TLSI**

| **TLSI** | **Odds Ratio** | **Lower bound** | **Upper bound** | **p** |
| --- | --- | --- | --- | --- |
| Age | 1.002 | 0.969 | 1.037 | 0.896 |
| Male gender | 1.556 | 0.564 | 4.294 | 0.393 |
| Comorbidities | 1.348 | 0.921 | 1.972 | 0.124 |
| CRP | 0.999 | 0.993 | 1.006 | 0.844 |
| LDH | 1.002 | 0.997 | 1.007 | 0.508 |
| Hb | 0.881 | 0.693 | 1.121 | 0.304 |
| AST | 1.010 | 0.993 | 1.028 | 0.244 |
| Creatinine | 0.829 | 0.598 | 1.149 | 0.261 |
| D-Dimer | 1.000 | 1.000 | 1.000 | 0.138 |
| WBC | 1.034 | 0.851 | 1.257 | 0.737 |
| INR | 0.279 | 0.047 | 1.667 | 0.162 |
| IL-6 | 1.003 | 0.998 | 1.009 | 0.240 |
| N/L ratio | 0.944 | 0.840 | 1.060 | 0.327 |
| **TLSI** | **2.596** | **1.660** | **4.059** | **< 0.001** |

Comorbidities score: number of concomitant chronic disease(s); CRP: C-reactive protein; LDH: lactate dehydrogenase; Hb: hemoglobin; AST: aspartate transaminase; WBC: white blood cell absolute count; INR: international normalized ratio; IL-6: interleukin-6; N/L ratio: neutrophil-to-lymphocyte ratio. TLSI: T-lymphocyte subset index, representing the number of T-lymphocyte subset absolute counts under the cut-off value, ranging from 0 to 4.

Multivariable logistic regression analyses were performed. Odds ratios and 95% Confidence Interval (lower bound-upper bound) are reported.
